# Reproductive health equity: demystifying unmet need for family planning among young women in Uttar Pradesh

**DOI:** 10.1186/s40834-024-00335-2

**Published:** 2025-01-08

**Authors:** Punit Mishra

**Affiliations:** PopulationCouncil Consulting, New Delhi, 110003 India

**Keywords:** Unmet need, UP, Family planning, Adolescents, NFHS-5, Decomposition analysis

## Abstract

**Background:**

The unmet need for contraception among adolescent women in India is a significant public health concern, contributing to unintended pregnancies and abortions. This paper seeks to examine the regional variations and factors driving rural–urban disparities in unmet family planning needs in Uttar Pradesh (UP), India’s most populous state, where the total unmet need among adolescents is as high as 19%.

**Methods:**

The study is based on 11,018 adolescent women from the recent round of India’s DHS, NFHS-5 (2019–21). To fulfil the study’s objective, Multilevel logit model and Oaxaca Blinder Decomposition was applied.

**Results:**

The Multilevel logit model results show statistically significant variations at community and district level, implying a strong presence of unobserved factors affecting the unmet demand. Oaxaca decomposition results show that difference in rural and urban adolescent unmet need is explained by factors like wealth, religion and intra-state regions.

**Conclusion:**

The results call for the need to implement culturally appropriate reproductive and sexual health literacy programs to increase uniform access to modern contraception and to raise women’s autonomy in the state of Uttar Pradesh.

## Introduction

Lack of access to family planning services is a big concern in developing countries even in 21st Century. In India, about 12.8% of married women had an unmet need for family planning in 2005–06, and this varied across different regions of the country [1]. Approximately 23% of young married women between the ages of 15 and 24 required contraception but faced barriers in obtaining it [2]. The problem is particularly huge among teenagers and young women. In five low-income countries, the percentage of women needing assistance ranges from 25 to 96% six weeks following childbirth and the highest level of demand is observed among women under the age of 20 years [3]. In India, despite various government initiatives, the unmet need for family planning remains high, leading to unintended pregnancies, unsafe abortions, and maternal deaths. From 1993 to 2016, these efforts prevented 56 million unintended pregnancies, 7 million unsafe abortions, and 167,000 maternal deaths [4]. However, in the urban slums of Uttar Pradesh (UP), 55.3% of young married women (15–24 years old) still lack access to family planning services [5]. A study from Uttar Pradesh shows a substantial increase in unmet need for traditional methods, but there is still a high demand for modern family planning methods and regional variation can also be observed [6]. Another study provided some projection-based estimations and suggested that by 2030, Uttar Pradesh will need an additional 92 million users of modern contraception to achieve the target of 75 percent for family planning [7]. Currently, the estimated unmet need for family planning in Uttar Pradesh ranges from 4.5 million to 57.5 million users by 2025, with six districts showing high prevalence rates of modern contraceptive use [8]. Furthermore, there is a greater demand for family planning to limit childbearing compared to spacing births in all six cities of Uttar Pradesh [9]. In the urban areas of Uttar Pradesh, over 15% of married women have an unmet need for family planning [10].

It is important to examine the scale and reasons for unmet demand among adolescents because high level of unmet need for contraception results in unintended pregnancies. Because the pregnancies are unintended, two-thirds of these pregnancies end in childbirth while the other third end in abortion. However, since adolescents often lack access to legal and high-quality abortion services, many of the abortions carried out are unsafe which in severe cases also lead to death. There are various factors affecting unmet need of family planning. For instance, early marriage leads to unmet need of family planning due to multiple issues. In conservative societies, girls in early marriages, feel pressure to prove fertility and marriage worthiness [11]. The unmet need for family planning is primarily focused among poor individuals in rural Uttar Pradesh and major determining factors are household wealth and the gender composition of children [12]. Another study found around 30.5% prevalence of unmet need for family planning in married women, and is influenced by factors such as low literacy rates, lack of awareness about available methods, and a desire for more children [13].

Education also plays a critical role, as spending more time in school is a known determinant of higher chance of having greater knowledge about contraceptives. According to one study, each additional years of schooling, reduces fertility by 10 percent among girls [14]. Usage of contraceptive is stigmatized and due to lack of knowledge, condom usage is linked with lack of manhood, partner distrust, or sickness. Lack of knowledge about how the contraceptives operate also leads to unmet needs. Out of fear, shame or stigma, under-age couples do not consider accessing available options for family planning [11, 15]. Religion is another determining factor, where pregnancy is seen as God’s will and the adolescents do not want to interfere in God’s plan for them [16–18].

Literature about unmet needs of family planning among adolescents is limited in India. The estimated scale of unmet needs is underestimated because most studies exclude the age group of 15–24 years even after having clear evidence that members of these age groups do indulge in sexual behaviours, essentially ignoring half of the adolescent population. Total unmet need at 19 percent among adolescents is a major problem given the high population of the state [19]. Addressing the unmet need for family planning among adolescents in UP is further crucial for improving reproductive health outcomes and achieving national family planning targets. The current paper examines the recent trends, determinants, and intrastate differences in unmet needs of family planning services among married adolescents and young women aged 15–24 years in India’s most populous state, UP. The study is an attempt to explore the associated trends of unmet needs across the state and the factors responsible for regional variation in unmet needs. Understanding current progress in family planning, identifying the factors that need focus to sustain the recent declining trend of unmet needs and predictors leading to rural urban differences in unmet need for family planning in UP is highly imperative research from policy and program perspective.

## Methodology

### Data source

The paper used the latest round of Demographic and Health Survey (DHS) namely National Family Health Survey (NFHS) in India conducted during, 2019–21. The survey has been conducted by IIPS, Mumbai since early 90 s. Funding for NFHS-5 was provided by the MoHFW, Government of India. ICF, USA provided technical assistance through the DHS Program, which is funded by USAID. The survey has gathered the wide range of demographic and health related information, including fertility, family welfare, reproductive health, mortality, nutrition, sexual behaviour, domestic violence, HIV prevalence etc. It has also been aptly designed to evaluate the success of Family Planning Program in India and its States. The fifth round of survey collected data from 636,699 households; 724,115 women of reproductive age groups (15–49 years); and 101,839 men spread over 707 districts spanned over 28 states and 8 Union Territories of India. Further to make a comparison from NFHS-1 to NFHS-5 [19–22], first four rounds of data have also been utilised. The sample size for the study was taken from women’s file with focus on Uttar Pradesh (UP) which consisted of young women (aged 15–24) who were fecund and currently married or cohabiting and had complete cases for all the variables of interest (*N* = 11,018).

### Study variables

#### Outcome variable

The outcome variable of interest is Unmet Need of Contraception, divided into two parts, first is Unmet need for spacing defined as: the proportion of fecund and sexually active women who want to delay their next pregnancy but are not using any method of contraception, and pregnant and amenorrhoeic women whose current pregnancy/birth were mistimed, and they were not using contraceptive method at the time they conceived. The second is Unmet need for limiting, this group represents the fecund and sexually active women who desire to limit the childbirth but not using any contraceptive method, also pregnant and amenorrhoeic women with unwanted birth/pregnancy who were not using any method before conceiving [23, 24]. The sum of unmet need of spacing and limiting is termed as the Total Unmet Need.

#### Independent variables

The explanatory variables were individual socioeconomic and demographic characteristics such as place of residence (urban/rural), wealth index divided into five quantiles, religion consist two groups namely Non-Muslim comprised Hindu Christians, Sikhs, Buddhist/Neo-Buddhist, Jain, Jewish, Parsi/Zoroastrian Donyi polo and others and Muslims, caste classed in Schedule Caste (SC)/ Schedule Tribe (ST), Other Backward Caste (OBC) and Others, this stratification uses the terminology adopted by the Government of India to focus more on socially deprived castes or tribes. These social groups are classified as SC, ST and OBC. Media exposure was divided into three categories no exposure: not exposed to any kind of media (radio/television/newspaper), partial exposure: exposed to any one kind of media and full exposure: exposed to all three kind of media, occupation categorised into five groups, educational level of women was categorised as illiterate, primary educated, secondary educated and higher, age at marriage of respondents grouped into three categories married in less than 14 years, 15–19 years and 20–24 years, current age of respondent divide into two groups (15–19 and 20–24), number of living children categorised as-at most one child, two children, three children and four & above children. Sex preference comprised of three sub-parts viz. no preference, preference for son and preference for daughter and women empowerment which is a composite indicator deduced from two variables: first one is sole, joint and no participation of women in three household decisions of own healthcare, purchase of large items and visit to friends and family, which was further categorised into three groups full, partial and no decision making power. Experience of domestic violence was dichotomized as yes or no. These variables are selected based on their significance in previous studies. Seventy-one districts of UP were covered for data collection in NFHS-5, to examine the regional pattern of unmet need, these districts were recognised into five regional zones, namely northern upper Ganga, central, eastern, southern and southern upper Ganga.

### Statistical analysis

The data were analysed at the univariate, bivariate, and multivariate levels to identify important predictors governing the unmet demand for contraception. Prevalence of unmet contraceptive need for spacing, limiting, and total unmet need among the young women was described at univariate level. While unmet need by respondent characteristics were described using percentage at bivariate level. At the multivariate level, Three Level Binary Logistic Regression Model was employed based on hierarchy of influence on unmet demand of contraception, namely individual and community level. The regression was used to examine the differences offered by different layers of explanatory variables on the study variable, and to show the effects that vary by community. In this model individual young woman was regarded as level one, cluster as level two and, districts of UP as level three. We were thus able to consider the effect of both sample clustering and unobserved factors at community level. Let us define the model we estimated in the analysis;

1 $$log\frac{\left({p}_{kji}\right)}{\left({1-p}_{kji}\right)}= \alpha +{X}_{kji}\beta +{\mu }_{kj}+{\delta }_{k}$$

Where, subscript $k,j,i$ denote district, cluster and women respectively.

${p}_{kji}$ is the probability of i-th women of community j and district k reported unmet need for contraception:

$\alpha$ the constant corresponding to study variable.

${X}_{kji}$ the covariates for women i for community j and district k, including socioeconomic and demographics characteristics;

$\beta$ the vector of parameters to be estimated corresponding to the socioeconomic, demographic factors at the individual level;

${\mu }_{kj}$ the random effect at cluster level within district k; and;

${\delta }_{k}$ the random effect at the district k level.

Both ${\mu }_{kj}$ and ${\delta }_{k}$ are random variables at cluster and district level and assume to follow multivariate normal distributions with mean 0 and variances unit. To examine the contribution of various factors on the differences present in unmet need for contraception due to the place of residence, multivariate decomposition analysis also known as Oaxaca Blinder decomposition analysis [25] was used. This method has frequently been applied to analyze gender differences and various other types of differences. It has also gained popularity in health disparities research hence, as per the available data and our study’s need, this method of decomposition seemed appropriate to study the variation between the two regions. This technique was first designed for linear regression model and later extended to nonlinear models [26]. This approach provides a scope to separate the difference into two parts; namely endowment (explained) and coefficient (unexplained). Endowment is the component explained by the change in the variable, whereas the coefficient is the factor computed by the change in the composition of variable. For example, in the current study unmet need is the study variable and women’s education is a factor associated with unmet need and the difference in unmet need is decomposed over the place of residence by education level, then explained part is the amount contributed by the difference in the women by their education level and unexplained part is the amount contributed by the difference in the effect of education level. The decomposition analysis utilizes two pieces of information: the prevalence of unmet need for all selected covariates for both Urban and Rural parts of UP and the coefficient derived from multivariate binary logistic regression model predicting unmet demand for contraception for both the dwelling setting separately. STATA 15.0 was used to perform all analyses and appropriate sampling weights were used in the estimation procedure.

## Results

The Levels and Trends in Unmet Needs of Young Women: The National and State Level Scenario. The prevalence of unmet contraceptive need in each of the five surveys for India and UP, and percentage change in successive surveys are computed and presented in the Table 1. The unmet need for spacing has shown a steady decline from NFHS – I through NFHS – V while the unmet need for limiting has gone up over the years, both at the national level as well as at the state level. Between 1992–93 and 2019–21, the unmet needs for spacing have declined by 57.4 per cent at the national level and by 58.8 per cent at the state level, while the unmet need for limiting have gone up by 2.8 per cent at the national level and by 46.0 per cent at the state level. The total unmet needs have shown a steady decline at both the levels; however, this decline is majorly driven by the decline in unmet need for spacing.

**Table 1:** Trends in unmet need for FP among adolescens and young women in India and Uttar Pradesh, 2019–21

	NFHS-1	NFHS-2	NFHS-3	NFHS-4	NFHS-5	**Percentage change in the unmet need for FP**				
						NFHS-1 to NFHS-2	NFHS-2 to NFHS-3	NFHS-3 to NFHS-4	NFHS-4 to NFHS-5	NFHS-1 to NFHS-5
**India**										
Spacing	27.1	20.8	17.6	16.5	11.5	-23.4	-15.4	6.0	-30.1	-57.4
Limiting	3.9	4.8	5.6	5.7	4.0	21.6	16.3	3.2	29.6	2.8
Total	31.1	25.6	23.1	22.3	15.6	-17.7	-9.5	-3.8	-30.3	-50.0
**Uttar Pradesh**										
Spacing	35.0	25.3	22.9	19.3	14.4	-27.6	-9.6	-15.7	-25.2	-58.8
Limiting	4.0	6.4	7.4	5.9	4.2	59.4	15.0	19.9	28.6	5.3
Total	39.0	31.7	30.3	25.2	18.6	-18.6	-4.6	-16.8	-26.0	-52.2

Source: Estimated from various rounds of NFHS

**Fig. 1:**
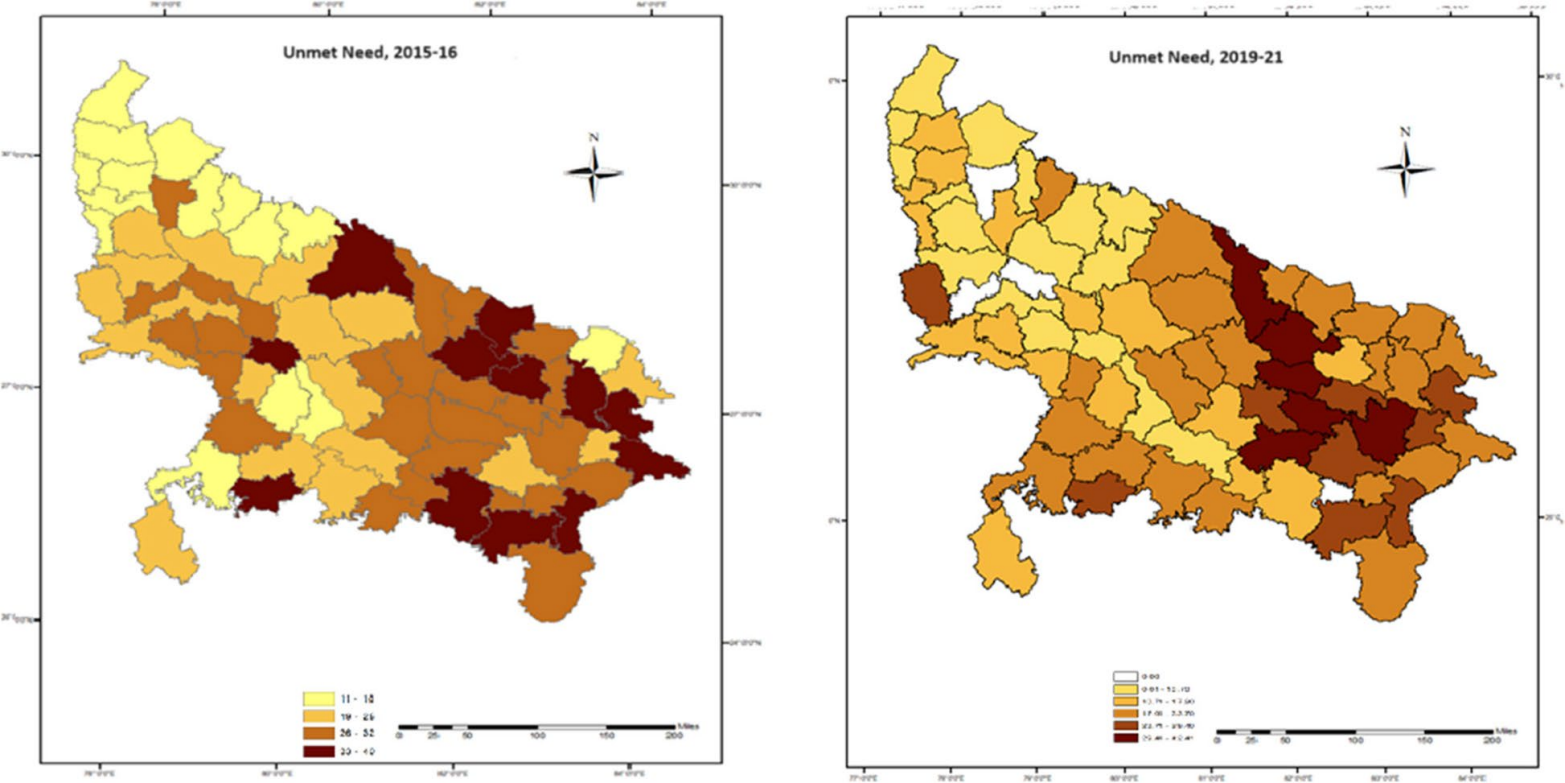
Spatial distribution of unmet need for FP among adolescent and young women in Uttar Pradesh, 2015–16 and 2019–21

**Fig. 2:**
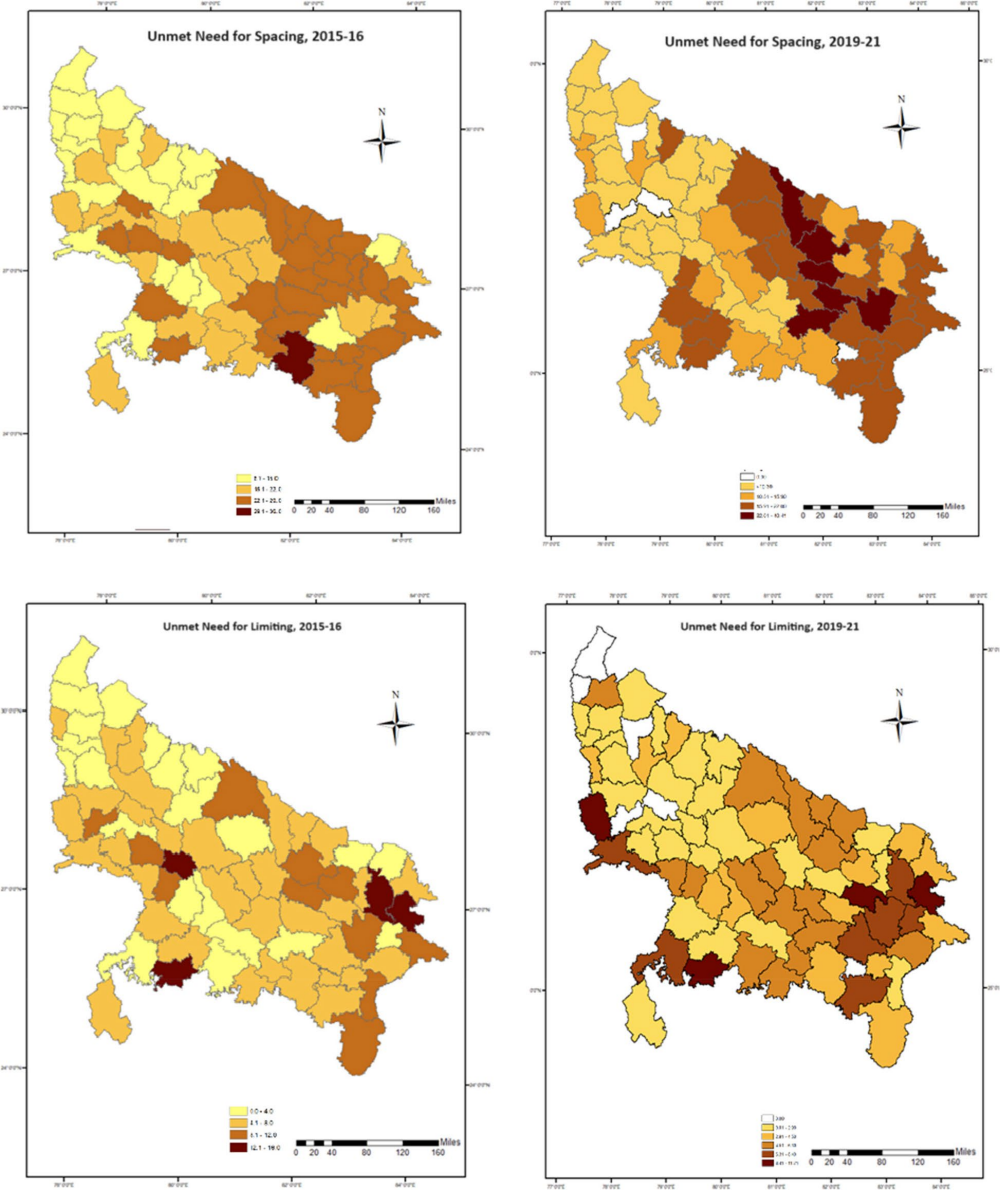
Spatial distribution of unmet need for spacing and limiting for FP among adolescent and young women in Uttar Pradesh, 2015–16 and 2019–21

The regional distribution of unmet need for contraception among the adolescent women are shown in Figs. 1 and 2. Eastern region has the highest unmet need for FP in all three categories, followed by central region; which registered a 7-point decline in unmet need for spacing from eastern zone, while only 2-point decrement is noted down in unmet need for limiting. Regional variations for unmet need for spacing were much higher; which ranged from 11 percent (northern upper ganga) to 25 percent (eastern) compared to the unmet need for limiting; varying from 3 to 8 percent. However, it is evident from the maps (Figs. 1 and 2) that, the adolescent women residing in southern and southern upper Gangetic regions of Uttar Pradesh offered minimal variation (less than one point) in unmet demand of contraceptive methods either for spacing or limiting.

Within the state, at the district level, there are large variations in the total unmet needs as well as unmet needs for spacing and limiting. As far as total unmet needs are concerned, Gorakhpur (eastern region) has reported maximum total unmet need, while Meerut comes under the northern upper ganga zone has reported minimum total unmet need. Allahabad has reported maximum unmet need for spacing while Kanpur Dehat has reportedly highest unmet needs for limiting. It is interesting to mention that; adolescent women residing in Rampur district recorded zero unmet demand for contraceptive to limit their next conception. On the other hand, the same district registered the highest unmet demand for spacing among all 10 districts of northern upper Gangetic region. Evidently, the unmet need for spacing in all 29 districts of eastern region are higher than the state level prevalence.

The prevalence of unmet need for spacing, limiting and combine among the adolescent women of UP according to the selected socioeconomic and demographics covariates is shown in Table 2. As evident from the table that young women living in rural parts of the state had greater proportion of unmet need both for spacing and limiting. Besides dwelling area, socio-religious divisions also play a crucial factor in regulating the unmet need for contraception, STs and Non-Muslim women have significantly higher unmet need for contraception than their counterparts. Those who were exposed to all three forms of media had highest proportion of unmet demand for contraception than their counterparts. A monotonic decreasing association was noted between the education of women and unmet demand for contraception, illiterate women had highest unmet need which gradually decreased with increasing level of education. A similar inverse association is also manifested between the household’s wealth status and unmet need for contraception. Women with white collar jobs (clerical, managerial, professional, technical) had greatest proportion of unmet need for spacing whereas women engaged in agricultural works showed greatest proportion of unmet need for limiting.

**Table 2:** Socioeconomic indicators and unmet need for FP among adolescent and young women in Uttar Pradesh, 2019–21

*N* = 11018	Currently Women age group 15–24								
**Background Variables**		**CI (95%)**			**CI (95%)**			**CI (95%)**	
	**Spacing**	**LL**	**UL**	**Limiting**	**LL**	**UL**	**Total**	**LL**	**UL**
**Place of residence**									
Urban	11.6	10.4	13.0	2.8	2.2	3.6	14.4	13.1	15.9
Rural	15.0	14.4	15.7	4.5	4.1	4.9	19.5	18.8	20.2
**Wealth status**									
Poorest	16.5	15.2	17.8	4.8	4.1	5.6	21.2	19.8	22.7
Poorer	14.7	13.6	15.9	5.1	4.5	5.9	19.8	18.6	21.1
Middle	14.9	13.6	16.3	3.5	2.9	4.3	18.4	17.1	19.9
Richer	13.0	11.6	14.4	4.0	3.3	5.0	17.0	15.5	18.6
Richest	11.6	10.2	13.1	2.6	2.0	3.5	14.2	12.7	15.9
**Religion**									
Non-Muslim	14.6	14.0	15.3	4.4	4.1	4.8	19.1	18.4	19.8
Muslim	13.3	11.9	14.8	3.0	2.3	3.8	16.3	14.7	17.9
**Caste**									
SC	14.5	13.4	15.7	3.7	3.2	4.4	18.2	17.0	19.5
ST	15.2	11.1	20.4	3.3	1.6	6.5	18.5	14.0	24.0
OBC	15.0	14.2	15.8	4.5	4.1	5.0	19.4	18.6	20.4
Others	12.5	11.1	13.9	4.2	3.4	5.1	16.6	15.1	18.2
**Media exposure**									
No	15.3	14.3	16.4	4.1	3.5	4.7	19.4	18.2	20.6
Partial	13.5	12.7	14.3	4.0	3.6	4.5	17.5	16.6	18.4
Full	16.0	14.4	17.7	5.2	4.3	6.3	21.2	19.5	23.1
**Occupation**									
Not working	16.4	14.8	18.1	4.3	3.5	5.3	20.7	18.9	22.6
Working	14.7	10.9	19.5	2.6	1.3	5.5	17.3	13.2	22.4
Don’t know/missing	14.1	13.5	14.8	4.2	3.9	4.6	18.3	17.6	19.1
**Education**									
Illiterate	13.8	12.5	15.3	3.9	3.1	4.7	17.7	16.2	19.3
Primary	12.1	10.6	13.8	4.5	3.6	5.7	16.6	14.9	18.5
Secondary	14.6	13.8	15.4	4.3	3.9	4.8	18.9	18.1	19.8
Higher	16.2	14.7	17.7	3.9	3.2	4.8	20.0	18.4	21.7
**Age at Marriage**									
< 14 years	13.4	11.2	16.0	7.4	5.7	9.4	20.8	18.1	23.8
15–19 years	13.7	13.0	14.4	5.0	4.5	5.5	18.7	17.9	19.5
20–24 years	16.0	14.9	17.1	2.1	1.7	2.6	18.1	17.0	19.3
**Age group**									
15–19	16.2	14.5	18.1	2.5	1.8	3.4	18.7	16.9	20.7
20–24	14.2	13.6	14.8	4.4	4.1	4.8	18.6	17.9	19.3
**Number of living children**									
At most one	15.7	15.0	16.4	1.8	1.6	2.1	17.5	16.8	18.2
Two children	11.3	10.1	12.6	11.0	9.9	12.3	22.3	20.7	23.9
Three Children	6.0	4.3	8.4	17.0	14.0	20.5	23.1	19.6	26.9
Four & above	2.1	0.4	9.6	17.0	10.0	27.3	19.0	11.6	29.6
**Sex preference**									
No preference	14.0	13.3	14.6	4.2	3.9	4.6	18.2	17.5	18.9
Son preference	16.4	15.0	17.9	4.0	3.3	4.8	20.4	18.9	22.1
Girl preference	15.8	11.4	21.5	4.7	2.5	8.7	20.5	15.5	26.6
**Women empowerment**									
Full decision & no violence	15.3	13.1	17.8	3.6	2.5	5.0	18.9	16.4	21.6
Partial decision & no violence	10.9	7.2	16.2	2.4	1.0	5.9	13.3	9.2	18.9
No decision & no violence	14.2	13.6	14.8	4.2	3.9	4.6	18.4	17.7	19.1
Full decision & violence	18.7	15.5	22.3	4.9	3.4	7.2	23.6	20.1	27.5
Partial decision & violence	16.4	11.4	23.2	7.2	4.1	12.6	23.7	17.6	31.1
No decision & violence	17.9	12.5	25.0	1.7	0.5	5.7	19.6	14.0	26.9
**Region**									
Northern upper Ganga	9.7	8.5	11.0	1.9	1.4	2.6	11.6	10.3	13.1
Central	14.1	12.6	15.7	3.8	3.0	4.7	17.9	16.2	19.6
Eastern	19.9	18.8	21.0	5.3	4.8	6.0	25.2	24.1	26.4
Southern	14.8	12.5	17.4	5.1	3.8	6.9	19.9	17.3	22.8
Southern upper Ganga	9.5	8.6	10.5	3.9	3.3	4.6	13.4	12.3	14.5

Source: Estimated from NFHS-5, 2019–21

*OBC* Other Backward Castes, *SC* Scheduled Castes, *ST* Scheduled Tribes

It is further noted that women experienced domestic violence and have no decision-making power had highest prevalence of unmet need for spacing, whereas the proportion of unmet need for limiting was highest among the women who had partial decision-making power and never experienced any form of domestic violence. Further, with rise in age at marriage the percentage of unmet need goes down, women married after 20 years of age experienced 80 percent and 30 percent lower unmet need for limiting and Total than to those women who were married at less than 14 years of age, except for unmet need for spacing which seemed to slightly increase (less than one point) from age at marriage 15–19 years to 20–24 years. In terms of age, young women aged 15–19 had the lowest proportion of unmet demand for limiting and for total compared to those aged 20–24. Use of contraceptive was directly driven by the woman’s current fertility status and her preference of a particular sex in child. It is evident from the table that women with higher order of birth have lower unmet demand for spacing and higher unmet demand to limit the childbirth. Women who prefer son over daughter also have lower unmet need for spacing and higher unmet need for limiting.

The determinants of unmet need for FP among young and adolescents in Uttar Pradesh is depicted in Table 3. The impact of movement from one sociodemographic stratum to another on the unmet demand for contraception has been worked out through three level binary logistic regression. The regression result in terms of log odds is reported in the table. The socio-economic factors, place of residence, literacy level and household wealth status, all three, have significant effect on unmet need for contraception, whereas occupation of young women doesn’t have significant impact on the same. Results showed that the risk of experiencing unmet need for FP is greater (almost 16 percent) if a woman lives in rural areas of UP rather than the urban areas and is lower (25 percent) if they belong to the richest wealth category. Women’s education attainment shows significant odds; the direction of causality indicates a monotonic increase in the unmet need for contraception as the education of women increase. Notably, the chance of unmet need is nearly 16 percent lower among the women who were partially exposed to any form of media compared to women with no media exposure. Women empowerment was insignificant when controlled with other covariates.

**Table 3:** Multilevel logistic regression: determinants of unmet need for FP among adolescent and young women in Uttar Pradesh, 2019–21

**Background Variable**	Odds Ratio	Std. Err	*P* > z	95% CI	
				Lower	Upper
**Place of residence**					
Urban	ref.				
Rural	1.188	0.117	0.080	0.980	1.441
**Wealth status**					
Poorest	ref.				
Poorer	0.902	0.067	0.168	0.779	1.044
Middle	0.900	0.077	0.218	0.761	1.064
Richer	0.901	0.089	0.294	0.742	1.094
Richest	0.755	0.094	0.023	0.592	0.962
**Education**					
Illiterate	ref.				
Primary	0.959	0.100	0.687	0.782	1.176
Secondary	1.159	0.094	0.070	0.988	1.360
Higher	1.213	0.131	0.074	0.982	1.499
**Occupation**					
Not working	ref.				
Working	0.635	0.143	0.044	0.408	0.988
Don’t know/missing	0.700	0.138	0.070	0.476	1.030
**Age group**					
15–24	ref,				
25–34	0.896	0.079	0.212	0.754	1.065
**Number of living children**					
At most one	ref.				
Two children	1.419	0.098	0.000	1.238	1.625
Three Children	1.505	0.204	0.003	1.154	1.963
Four & above	1.205	0.442	0.612	0.587	2.474
**Age at Marriage**					
< 14 years	ref.				
15–19 years	1.048	0.117	0.677	0.842	1.304
20–24 years	1.129	0.141	0.331	0.884	1.442
**Caste**					
SC/ST	ref.				
OBC	0.951	0.088	0.589	0.793	1.140
Others	1.121	0.081	0.113	0.973	1.292
**Religion**					
Non-Muslim	ref.				
Muslim	0.888	0.078	0.176	0.747	1.055
**Media exposure**					
No	ref.				
Partial	0.844	0.042	0.001	0.765	0.930
Full	0.943	0.083	0.502	0.794	1.120
**Sex preference**					
No preference	ref.				
Son preference	1.044	0.072	0.530	0.912	1.195
Girl preference	0.953	0.197	0.815	0.635	1.430
**Women empowerment**					
Full decision & no violence	ref.				
Partial decision & no violence	0.751	0.192	0.264	0.454	1.241
No decision & no violence	1.307	0.287	0.223	0.850	2.011
Full decision & violence	1.196	0.201	0.288	0.860	1.664
Partial decision & violence	1.322	0.338	0.276	0.800	2.183
No decision & violence	0.904	0.243	0.708	0.534	1.530
Constant	0.164	0.036	0.000	0.107	0.251
LR test vs. logistic model: chi2(2)	245.660				
Prob > chi2	0.000				
Wald chi2(28)	70.99				
**Random-effects Parameters**					
District (SD)	0.506	0.054	0.000	0.410	0.623
Cluster (SD)	0.538	0.058	0.000	0.435	0.665

Source: Estimated from NFHS-5, 2019-21

*CI* Confidence Interval, *OBC* Other Backward Castes, *ref.* reference category, *SD* Standard Deviation, *SC* Scheduled Castes, *ST* Scheduled Tribes

Demographic factors also played a significant role in the unmet demand for FP. Women’s age at marriage significantly increased the probability of unmet demand for contraception at marital ages 15–19 and 20–24 years relative to those married before age 14. Increasing parity led to higher odds for unmet need, women with two or more than two children had a higher likelihood of unmet need for contraception compared to nulliparous women or women who had one child. Sex preference in fertility was not a significant factor of unmet need, except marginally where women having son preference relative to those with no sex preference were 4 percent more likely to report an unmet need for contraception.

The Random effect model is used to measure the unobserved heterogeneity present in the data and the computed variations at both cluster and district level in terms of standard deviation (SD) are given in Table 3 At the cluster and district level statistically significant random disturbance terms are found, this strongly suggested a presence of some unobserved factors affecting the unmet demand of contraception. There may be unobserved components related to family planning service facility such as availability of different methods, quality of service, reach to facility centre, as well as social development at community (cluster or district) level, all these jointly effect the use of family planning and offered a significant variation in measuring the unmet demand for contraception either for spacing or limiting. Lastly, the significant value of likelihood ratio test confirmed that the model adequately fitted.

Unmet demand of contraception is a major concern in the state of Uttar Pradesh. Table 4 addresses the variation present in the unmet demand of contraception due to the women’s place of residence. The decomposition further bifurcated this difference into two components, i.e., explained part which measures the gap attributable to the difference in women characterises (urban vs rural) and unexplained part which reports the effects of these characteristics and their interaction with characteristics. It is evident from the table that young and adolescent women who lived in rural parts of UP experienced greater unmet demand for contraception than women who lived in urban areas. Of the total difference in unmet need over the group, almost 55.2 percent was accounted by endowment effects which can be explained by the change in the composition of women. Remaining 44.8 percent unexplained difference was driven by coefficient effects of selected explanatory variables and the interaction term, but the result is insignificant at 5 percent level of significance.

**Table 4:** Oaxaca-blinder decomposition: Urban–Rural difference in unmet need for FP among adolescent and young women in Uttar Pradesh, 2019–21

Group Variable	Coef	*P* > z	95% CI	
			**Lower**	**Upper**
Urban	14.55	0.000		
Rural	19.14	0.000		
Difference in unmet need	-4.58	0.000		
Difference explained	55.19	0.000		
Difference unexplained	44.81	0.038		
**Background variable**				
**Explained difference**				
**Wealth status**				
Poorer	-8.05	0.266	-41.46	4.19
Middle	-1.09	0.506	-8.03	1.45
Richer	2.01	0.768	-21.21	10.53
Richest	26.99	0.131	-14.99	42.38
**Religion**				
Muslim	6.31	0.282	-9.68	12.18
**Caste**				
OBC	0.01	0.975	-1.69	0.64
Others	0.25	0.889	-6.14	2.60
** Occupation**				
Working	-0.52	0.500	-3.79	0.68
Don’t know/missing	-1.53	0.420	-9.75	1.49
**Education**				
Primary	-0.34	0.735	-4.36	1.13
Secondary	2.42	0.143	-1.53	3.87
Higher	-7.53	0.086	-30.07	0.73
**Media exposure**				
Partial	4.97	0.137	-2.95	7.87
Full	0.16	0.799	-1.97	0.94
**Age at Marriage**				
15–19 years	2.37	0.536	-9.59	6.76
20–24 years	-6.97	0.236	-34.47	3.12
**Age group**				
20–24	1.94	0.273	-2.85	3.70
**Number of living children**				
Two children	1.90	0.306	-3.24	3.78
Three children	3.06	0.026	0.68	3.94
Four & above	0.18	0.590	-0.88	0.57
**Sex preference**				
Son preference	-0.05	0.961	-4.23	1.48
Girl preference	0.00	0.994	-0.86	0.31
**Women empowerment**				
Partial decision & no violence	0.12	0.789	-1.46	0.71
No decision & no violence	0.98	0.485	-3.31	2.56
Full decision & violence	0.72	0.477	-2.36	1.85
Partial decision & violence	0.31	0.480	-1.01	0.79
No decision & violence	0.03	0.839	-0.51	0.23
**Region**				
Central	-3.37	0.108	-13.93	0.51
Eastern	81.36	0.000	102.93	73.45
Southern	-5.03	0.087	-20.10	0.50
Southern upper Ganga	-1.62	0.272	-8.42	0.87

Source: Estimated from NFHS-5, 2019–21

*CI* Confidence Interval, *Coef* Coefficient, *OBC* Other backward castes

As the explained component of difference was much higher than the unexplained factor; the contribution of independent variables was also computed and presented in Table 4. The explained difference varied substantially from one variable to another and according to the categories within variable. Change in the proportion of women having son preference contributed to a reduction in unmet need for contraception among the urban women, whereas the difference in the composition of women having three children contributed greatest increment (around 42 percent) in unmet demand for contraception between the two dwelling settings urban vs rural. All other socioeconomic and demographics variables considered in the study: religion, caste, media exposure, education, wealth, occupation, women empowerment age at marriage and current age of women, failed to show a significant contribution to explain the urban–rural differences that exist in unmet need for contraception.

## Discussion

This paper was an effort to examine the socioeconomic and demographic factors associated with the unmet contraceptive need both for spacing and limiting in Uttar Pradesh. The current study not only provided additional information on the trends and patterns of unmet need but has also made three key contributions to the family planning literature. Firstly, the study is focused on Uttar Pradesh, which stands out as the most populous division of India, constituting 17 percent of total population. Undeniably, the contraceptive use dynamics in this highly populous state is of utmost concern both to the policy makers and demographers in India. The importance of conducting a study concerning UP because of its population size and unmet need was also discussed by other literature sources [27, 28]. Secondly, the current study extended the search for significant correlates of unmet contraceptive need among young and adolescent women by examining the individual and community characteristics that may shape the level of unmet need for family planning in UP. Lastly, the study addressed the factors responsible for urban–rural differences in unmet need for FP and provided information on wider range of covariates which can be targeted by policy makers in the state of UP.

The results show that UP has made significant progress in satisfying the unmet need for family planning among adolescents and young women, still it has a long way to go, especially in case of unmet needs for limiting. The total unmet need in the state has declined over the course of five rounds of National Family and Health Survey, however, this decline is mainly due to a steady decline in the unmet needs for spacing. The unmet needs for limiting have gone up over the considered time-period. More than a quarter of young married women were having an unmet demand for FP of which, about 24 percent was for limiting and rest 76 percent for spacing. This is almost 13 percent higher than the total unmet need reported at national level; this greater unmet demand is majorly due to the higher unmet demand to space the birth than to limit it among the adolescent of UP. The prevalence of unmet need is also higher than that reported in rural Uttar Pradesh by NFHS-5 [21]. Education as expected is a key determinant of contraceptive usage [17]. The regional pattern of unmet need for contraception among the young married women of Uttar Pradesh shows that Eastern region occupies the highest total unmet need as well as unmet need for spacing and limiting followed by Central and Southern region. This regional variation studied through maps shows varying district level prevalence of unmet need, the districts with high female literacy rate (70 percent or above) such as Gautam Buddha Nagar, Ghaziabad, Kanpur Nagar, Lucknow etc. recorded less unmet demand except for Etawah where the total unmet need for contraception was recorded higher than the state average. Whereas Eastern region which has some of the most developed districts like Varanasi, Kushinagar, Allahabad offered almost 6 to 14 point higher unmet demand for FP than that computed at state level. This variation can be attributed to various socioeconomic cofactors which were also reported in another study which discussed about women’s socioeconomic and demographic status playing a vital part in the adoption of FP [29]. The regression analysis also elicited significant and expected directions of effects of the selected socioeconomic and demographic predictors on the unmet demand for contraception. As afore-discussed, women’s education leads to a significant and positive effect on unmet demand for contraception, the possible reason to account for this finding might be that young women with higher level of education have higher likelihood of postponing childbearing [30–32]. Furthermore, the study also unraveled that woman with higher parity (three or more children) experienced higher risk of unmet need which is in concurrence with studies done in developing countries that reported lower unmet need among nulliparous women [33]. Contrary to this, Shukla et al. [34], and Imasiku et al. [35] in their research found higher unmet contraceptive demands in nulliparous women. Preference for sons as child came out to be a significant determinant of unmet need which bolsters the widely prevalent concept of “Male child preference” in the study area. A similar finding was noted by Yadav et al. [5] in their study of Empowered Action Groups (EAG) states, women who preferred sons over daughters had lesser unmet need than their counterparts. An interesting observation of the present study was that respondents with preference for female child had 4 percent higher unmet contraceptive need as compared to those who had no preference.

Results of decomposition analysis showed that there is a higher unmet need in rural areas than urban part of state, which is attributable more to the change in the sample population than to the change in the characteristics. Significant contribution in composition have occurred for richest women with three children and have son preference. Previous research [36] has suggested that in rural Uttar Pradesh, women were less likely to receive maternal health services, and advice on family planning methods during ANC and PNC visits. The insignificant contribution of change in characterises of selected variables along with the above-mentioned anomalies reflect the lower impact of government family planning initiatives in rural parts of UP. Other studies discussing rural urban differentials also state that unmet need is higher among adolescent women wanting to avoid pregnancy who live in rural areas and who live in poorer households [17, 28].

The examination of regional variation using DHS dataset along with the use of strong methodology enhances the validity of this paper. However, the analysis is limited to the demand side factors and not being able to include the supply side factors such as family planning service availability and its quality which also affects the use of contraception. Furthermore, use of contraceptive is not only a woman’s job, but husbands also play an important role in the dynamics of contraceptive usage, their non-inclusion in the current study may not reflect the overall perspective of the couple regarding the use of family planning services. This can be further expanded in future studies undertaken in the state.

## Conclusion

The government of India has been continuously struggling to slow down the population growth in Uttar Pradesh because a large size and high growth rate of population can have enormous negative implications on social as well as economic development. The unmet demand of family planning may add an added burden of either undesired births or unsafe abortions, which hinder the government’s efforts. The paper identifies the problems of a vulnerable group i.e., adolescent women which must be addressed to achieve the desired goal of sustainable development. Formation of community-based peer system will provide an opportunity for holistic discussion about family planning methods. This community-based peer groups will help the young women to overcome embarrassment or hesitation and will also give them autonomy to avail FP services. Changing the mindset of young generation at an early stage by introducing sexual and reproductive health knowledge as the part of our routine education system could go a long way to motivating them to adopt contraceptive in future and subsequently follow a healthy fertility behavior. Place of residence has been explored as a crucial determinant of unmet need for contraception. Regional variation in family planning has also been observed. Rural UP faces not only the shortage of family planning services but also have certain social beliefs due to which the risk of unmet need has been found to be higher there. Community influencers and religious leaders need to engage males and sensitize about the pros of family planning services. Policy makers and demographers need to prioritize rural areas using newer curated policies and put more efforts to achieve the reduction in unmet need.

## Data Availability

This study is based on anonymous public-use datasets with no identifiable information about the survey participants. Survey data is available upon request on the official website of the IIPS at: https://rchiips.org/nfhs/data1.shtml.

## Abbreviations

ANC: Antenatal Care
CI: Confidence Interval
DHS: Demographic and Health Survey
EAG: Empowered Action Groups
FP: Family Planning
LL: Lower Limit
MoHFW: Ministry of Health and Family Welfare
NFHS: National Family Health Survey
OBC: Other Backward Caste
PNC: Postnatal Care
SC: Schedule Caste
SD: Standard Deviation
ST: Schedule Tribe
UL: Upper Limit
UP: Uttar Pradesh
USAID: United States Agency For International Development
UT: Union Territory
